# Presence and Virulence Characteristics of Shiga Toxin *Escherichia coli* and Non-Shiga Toxin–Producing *Escherichia coli* O157 in Products from Animal Protein Supply Chain Enterprises in South Africa

**DOI:** 10.1089/fpd.2021.0062

**Published:** 2022-06-10

**Authors:** Evelyn Madoroba, Keneiloe Portia Malokotsa, Cynthia Ngwane, Sogolo Lebelo, Kudakwashe Magwedere

**Affiliations:** ^1^ Department of Biochemistry and Microbiology, University of Zululand, KwaDlangezwa, South Africa.; ^2^ Bacteriology Section, Agricultural Research Council–Onderstepoort Veterinary Research, Onderstepoort, South Africa.; ^3^ Agricultural Research Council—Biometry Unit, Onderstepoort, South Africa.; ^4^ Agriculture and Life Sciences, University of South Africa, Florida, South Africa.; ^5^ Directorate of Veterinary Public Health, Department of Agriculture, Land Reform and Rural Development, Pretoria, South Africa.

**Keywords:** food safety, STEC and non-STEC O157, meat and meat products, conventional and RT-PCR, culture methods, “One Health”

## Abstract

Consumption of food that is contaminated with Shiga toxin–producing *Escherichia coli* (STEC) has been linked to serious foodborne disease outbreaks. Our aim was to provide a descriptive study on the presence and virulence factors of STEC and non-STEC O157 isolates recovered from 2017 diverse meat and meat product samples from all provinces of South Africa (*n* = 1758) and imported meat from South Africa's major ports of entry (*n* = 259). A cross-sectional study was undertaken to analyze raw intact meat, raw processed (nonintact) meat, and ready-to-eat (RTE) meat from cattle, game, sheep, pork, and poultry. Isolation was performed using International Organization for Standardization-based microbiological techniques, while detection and characterization were performed using real-time PCR (RT-PCR) and conventional PCR targeting the *stx_1_*, *stx_2_*, *eae*, and *ehxA* genes. A total of 28 of 1758 (1.59%; confidence interval [CI] 1.1–2) samples from the domestic market tested positive (*n* = 10 *Escherichia coli* O157:H7; *n* = 14 *Escherichia coli* O157: non-H7; and *n* = 4 non-O157 STEC), while 4/259 (1.54%; CI 0.4–4) samples from ports of entry tested positive for *Escherichia coli* O157:H7 based on RT-PCR. On average, diverse samples from domestic meat and meat products from cattle showed the highest number of positive samples (22/1758; 1.3%; CI 0.8–2). RT-PCR detected more positive samples (*n* = 32) compared with culture (*n* = 17). Sixteen different virulence factor combinations were observed. Our findings demonstrate a relatively low presence of diverse STEC strains along the meat value chain. To our knowledge, this is the first extensive report in South Africa to analyze STEC and non-STEC O157 from local and imported samples from many animal species. This is important as it reveals virulence factors in STEC strains circulating in meat and meat products in South Africa, which contribute to the risk of infection.

## Introduction

Foodborne pathogens are major causes of foodborne illnesses, hospitalizations, and deaths worldwide with huge social and economic implications. In South Africa, 327 outbreaks of foodborne diseases were reported to cause 11,155 illnesses and 49 deaths between January 2013 and December 2017 (Shonhiwa *et al.*, 2018). Bacteria such as nontyphoidal *Salmonella* spp., *Campylobacter* spp., *Listeria monocytogenes*, *Staphylococcus aureus*, and Shiga toxin–producing *Escherichia coli* (STEC) cause foodborne illnesses (Scallan *et al.*, 2011).

Shiga toxin *Escherichia coli* comprise a diverse group of toxic strains that harbor *stx* genes and share a common theme of pathogenesis (FAO-WHO, 2019; Gonzalez-Escalona and Kase, 2019). Shiga toxin *E. coli stx_1_*, *stx_2_*, and associated virulence factors are linked to symptoms of STEC-induced infections (Yang *et al.*, 2020). In addition to Shiga toxins, intimin and hemolysin (*ehxA* genes) are important STEC virulence factors. The risk of severe illness from STEC infectivity may be predicted based on virulence factors identified for an STEC strain with illnesses such as bloody diarrhea, which may progress to life-threatening hemolytic uremic syndrome (HUS) (Gonzalez-Escalona and Kase, 2019). It has been postulated that production of *stx* alone without adherence may be insufficient for STEC to cause severe illness (FAO-WHO, 2019).

Diverse food sources are associated with STEC infections, but contaminated, undercooked ground beef has been reported to be a predominant vehicle for transmitting STEC infections (Arthur *et al.*, 2004; Erickson and Doyle, 2007; Onyeka *et al.*, 2020). Contamination may start at the farm level through shedding of contaminated feces by apparently healthy animals and during transportation of animals to the market (Barham *et al.*, 2002), during lairage to dressing or slaughter to dressing (Nastasijevic *et al.*, 2008), or at the retail level (Nastasijevic *et al.*, 2009) or consumer level (Bolton and Maunsell, 2004).

Different studies were undertaken to determine the extent of meat contamination by STEC (Kijima-Tanaka *et al.*, 2005; Barlow *et al.*, 2006; Ateba *et al.*, 2008, 2011; Fegan *et al.*, 2009; Ojo *et al.*, 2010; Akanbi *et al.*, 2011; Magwedere *et al.*, 2013; Onyeka *et al.*, 2020), but fragmented information exists on common zoonotic serotypes of *E. coli* (Magwedere *et al.*, 2013). In South Africa, there are few publications on STEC contamination of meat (Ateba and Mbewe, 2011; Onyeka *et al.*, 2020), which focused on single to few animal species from specified localities.

The aim of this study was to provide a descriptive study on the presence and virulence factors of STEC and non-STEC O157 in 2017 meat and meat product samples of beef, mutton, poultry, pork, and game from all nine provinces of South Africa (including imported meat from different countries at major ports of entry). To our knowledge, this is the first extensive descriptive study on the presence and virulence factors of STEC in meat and meat products from diverse animal species, meat types, and establishments in South Africa.

The findings are important to the international scientific community, policy makers, academics, food business owners, and consumers because food safety is a global issue that affects health and trade.

## Materials and Methods

### Study design

The cross-sectional study was undertaken based on production levels, distribution, and consumption patterns of different meat types in the nine provinces of South Africa where feasible. The sample size was based on convenience sampling, taking into consideration the European Food Safety Authority report on “Development of harmonized survey methods for foodborne pathogens in foodstuffs in the European Union” (Käsbohrer *et al.*, 2010).

Based on the equation and assuming a desired confidence level of 95% and prevalence of 50%, the required sample size is 384. As there are no data on the national prevalence of STEC in South Africa, we assumed a prevalence of 50%, as described by Käsbohrer *et al.* (2010).

$$n_{\infty }=\frac{{\left(Z_{\alpha }\right)}^{2}*p*\left(1-p\right)}{L^{2}}$$

where *n* = number of samples,

*p* = expected prevalence,

Z_α_ = desired confidence level set at 95% corresponding to 1.96, and

L = level of accuracy at 5%.

A larger convenience sample size of 2017 (1758 samples from meat produced in South Africa and 259 samples from imported meat) was used to minimize potential confounding and to enhance robustness. The sample information is shown in Table 1. It is important to note that the number of samples across types and provinces varied because convenience samples were collected.

**Table 1.** Imported Meat Samples and Samples from South Africa That Were Analyzed for the Presence of *Escherichia coli* O157:H7, *Escherichia coli* O157: Non-H7, and Non-O157 Shiga Toxin–Producing *Escherichia coli* Contamination

Sample type	Raw intact	Raw processed^a^	RTE^b^	Total
Meat produced in South Africa				
Beef	68	689	320	1077
Lamb/mutton	61	1	6	68
Poultry	329	14	57	400
Pork	83	20	32	135
Game	12	6	15	33
Mixed	4	35	6	45
Total	557	765	436	1758
Imported meat samples				
Poultry^b^	0	1	1	198^b^
Pork	0	0	0	11
Mixed^b^	0	0	0	5^b^
Total	0	1	1	259

^a^ Raw processed meat refers to nonintact meats such as ground/mince, patties, and sausage meats.

^b^ The meat categories for two of the imported poultry samples and 1 mixed meat sample were not specified, hence they are not included in the categories, but only under total numbers.

RTE, ready-to-eat.

### Sample collection and transportation

The samples were collected between October 2014 and December 2016, as described by Matle *et al.* (2019). Meat and meat products were collected from abattoirs (raw intact meat), processing plants, and retail outlets using sterile microbiological techniques. The samples were collected and transported according to recommendations in the “Standard for the microbiological monitoring of meat, process hygiene and cleaning” (Department of Agriculture Forestry and Fisheries, 2010).

Approximately 300 g of pooled meat or meat products was collected in sterile containers. Raw processed (nonintact) meats consisted of ground meat, hamburgers, or sausages. RTE meats were either polony or biltong. Samples were placed in cooler bags containing ice packs to ensure temperatures were maintained between 0°C and 4°C during transportation. All samples were analyzed using PCR screening of inoculated broths as well as culture in parallel.

### Microbiological analysis

*Escherichia coli* O157 and non-O157 *E. coli* were detected simultaneously using two approaches in parallel. *Escherichia coli* O157 was isolated as described in International Organization for Standardization 16654: 2001. The immunomagnetic coated bacterial cells (50 μL) were inoculated onto cefixime–tellurite–sorbitol MacConkey agar (CT-SMAC; Oxoid Limited, Hampshire, UK) and a second culture medium BBL CHROMagar O157 (Thermo Fisher, Paris, France), followed by incubation at 37°C for 18–24 h. The incubated CT-SMAC and CHROMagar O157 agar plates were evaluated and presumptive colonies were selected for further confirmation based on macroscopic appearance. Positive and negative controls were included alongside meat samples.

For non-O157 *E. coli*, samples were analyzed as described in the Bacteriological Analytical Manual with some modifications (Feng *et al.*, 2017). Meat samples (25 g) were macerated and 225 mL of Butterfield's buffer was added, followed by thorough mixing and direct streaking of the enriched samples onto STEC CHROMagar (Thermo Fisher) and MacConkey (Oxoid Limited) agars. Inoculated agar plates were incubated at 37°C for 18–24 h.

Presumptive *E. coli* isolates were identified using Gram stain, oxidase, and IMViC (indole production, methyl red, Voges–Proskauer, and citrate utilization) tests. All isolates that were oxidase and Gram-negative rods, indole positive, methyl red positive, Voges–Proskauer negative, and citrate negative were considered *E. coli*. The *E. coli* were then inoculated on blood tryptose agar (5% sheep blood) to evaluate hemolysis. Clear zones observed around bacterial cells were interpreted as beta hemolysis (β-hemolysis).

The latex agglutination test to distinguish *Escherichia coli* O157 from other non-O157 *E. coli* was done using Remel™ Wellcolex™ *Escherichia coli* O157:H7, according to the manufacturer's instructions (Life Technologies, Johannesburg, South Africa). There are two test reagents within the *Escherichia coli* O157:H7 kit, namely O157 and H7, which contain latex particles coated with specific antibodies for *Escherichia coli* O157 and *Escherichia coli* H7 antigens, respectively. All isolates were preserved in a mixture of nutrient broth containing glycerol (35% final concentration) in sterile Eppendorf tubes and stored at −20°C until required for further confirmation. All presumptive isolates were further confirmed by re-rerunning real-time PCR (RT-PCR).

### DNA extraction

Crude DNA for use in conventional PCR for determining virulence genes was extracted from pure cultures. The pure bacterial cells were suspended in sterile distilled water, and DNA for conventional PCR was extracted by boiling cells using the cell lysis method (Madoroba *et al.*, 2016).

The DNA for use in RT-PCR assays was extracted directly from portions of meat samples that were previously enriched in BPW (42 ± 1°C for 16–20 h; protocol B) using the Applied Biosystems™ PrepSEQ™ Nucleic Acid Extraction Kit, which involved manual extraction of nucleic acid as recommended by the manufacturer.

### Real-time PCR

RT-PCR assays for screening STEC and confirmation of STEC were verified in the laboratory to determine fitness for purpose through repeatability, reproducibility, specificity, and sensitivity assays using naturally contaminated samples and spiked meat samples. Negative and positive controls were included with each RT-PCR run (see section Reference strains for quality control).

All samples (*n* = 2017) were analyzed using MicroSEQ™ RT-PCR RapidFinder™ STEC screening and STEC confirmation (Thermo Fisher Scientific, Austin, TX, USA) (for STEC screen-positive samples) assays, which are validated by Association Française de Normalisation (AFNOR in French; French Standardization Association in English). The RapidFinder STEC screening assay is able to detect *stx_1_*, *stx_2_*, and *eae* virulence factors and *Escherichia coli* O157 (Cloke *et al.*, 2016).

The STEC confirmation assay is able to detect a group of 6 non-O157 STEC strains, which are commonly referred to as the “Big 6” as well as *Escherichia coli* O157:H7 (Cloke *et al.*, 2016). The MicroSEQ *Escherichia coli* O157:H7 Detection Kit (Applied Biosystems; Thermo Fisher Scientific) was used for detection of *Escherichia coli* O157:H7. Each reaction tube contained an internal positive control included by the manufacturer. Results were interpreted using RapidFinder Express Software.

### Conventional multiplex PCR

Virulence factors, *stx_1_*, *stx_2_*, *eae*, and *ehxA*, were amplified using multiplex PCR. The procedure for multiplex PCR was done according to the OIE Terrestrial Manual (2012) and modified by adding primers that target the *ehxA* gene (Paton and Paton, 2008). The fitness for purpose of the multiplex PCR was determined before use. Furthermore, a monoplex PCR was used for detection of *Escherichia coli* O157 by targeting the serotype-specific *rfbE* gene (Bertrand and Roig, 2007).

The final PCR volume of 25 μL comprised 12.5 μL of DreamTaq Green PCR Master Mix (2 × ) (Thermo Fischer Scientific), 10 μM of each primer (0.5 μL × 8 primers) (Inqaba Biotec, Pretoria, South Africa), DNA template (5 μL), and PCR water (3.5 μL). In addition, 16S rRNA PCR amplification was done for quality control using 27F and 1492R primers.

The tubes containing 25 μL of the PCR mixture were placed in a thermocycler (Eppendorf, Hamburg, Germany), followed by amplification using the following conditions: denaturation for 5 min at 96°C, 35 cycles of denaturation at 94°C for 30 s, annealing at 56°C for 30 s, and elongation for 1 min at 72°C. The final extension was undertaken at 72°C for 7 min. Molecular-grade water was used as the no-DNA control, and the positive controls are mentioned in the relevant section.

### Reference strains for quality control

Reference strains were always included with each batch of experiments for quality control purposes. *Escherichia coli* O157:H7 ATCC 43888 was used alongside *Escherichia coli* O157 field strains (including O157:H7 and O157:H^–^). Furthermore, non-O157 reference field strains in the culture collection of Feed and Food Analysis Laboratory, Bacteriology Section at Agricultural Research Council–Onderstepoort Veterinary Research (ARC-OVR), were used as positive controls for *stx_1_*, *stx_2_*, *eae*, and *ehxA* virulence factors.

*Escherichia coli* ATCC 25922 was the positive control for microbiological analysis and negative control for virulence factors. PCR water was used as the no-DNA control in all PCR experiments.

### Agarose gel electrophoresis

The PCR amplification products (for conventional PCR) were electrophoresed in 1.5% agarose gel stained with ethidium bromide at 3 V/cm for ∼1 h. A molecular weight marker (100 bp) was included in each gel for determining amplicon sizes. The stained agarose gels were visualized under ultraviolet light, and the results were captured using a gel documentation system (Bio-Rad, Hercules, CA, USA).

### Statistical analysis

Descriptive statistics were used for determination of the proportions of STEC-positive samples and virulence factors. The 95% binomial confidence limits were calculated using Excel (Snedecor and Cochran, 1967).

## Results and Discussion

Based on PCR results, the presence of contaminated meat was slightly higher in South Africa (28/1758; 1.59%; confidence interval [CI] 1.1–2) compared with imported meat (4/259; 1.5%; CI 0.4–4); however, it is possible that unlabeled or mislabeled imported meat placed on the domestic market could have been labeled as domestically produced meat. Interestingly, we incidentally detected 52 *Escherichia coli* O157:H7 isolates lacking all virulence factors (*eae*, *stx1*, *stx2*, and *ehxA*), but these unusual strains require further characterization, which is outside the scope of this article and will not be discussed further.

RT-PCR detected more positive *Escherichia coli* O157, *Escherichia coli* O157:H7, and non-O157 STEC compared with culture. *Escherichia coli* O157:H7, *Escherichia coli* O157: non-H7, and non-O157 STEC with 16 different gene combinations were observed from the culture-positive isolates.

Table 2 shows the distribution of meat and meat products that tested positive among different animal species and meat types. In general, raw processed (nonintact) meat (12/1758; 0.68; CI 0.4–1) and ready-to-eat (RTE) meat (13/1758; 0.74%; CI 0.4–1) constituted a relatively large proportion of positive samples from the domestic market compared with raw intact meat (3/1758; 0.17%).

**Table 2.** Summary of the Profiles of *Escherichia coli* O157:H7, *Escherichia coli* O157: Non-H7, and Non-O157 Shiga Toxin–Producing *Escherichia coli* and the Associated Animal Species and Meat Types for Positive Samples from South Africa

Isolate profile	Animal species					Meat category			Total
	Beef	Poultry	Lamb	Pork	Mixed meat	Raw	*p* ^a^	RTE	
*Escherichia coli* O157:H7/*eae*/*ehxA*	1	0	0	0	0	0	0	1	1
*Escherichia coli* O157:H7/*eae*/*stx1*/*stx2*/*ehxA*	0	1	0	0	0	0	0	1	1
*Escherichia coli* O157:H7/*eae*	2	1	0	0	0	0	0	3	3
*Escherichia coli* O157:H7/*stx1*	1	0	0	0	0	0	1	0	1
*Escherichia coli* O157:H7*/eae*/*stx2*	0	0	0	1	0	1	0	0	1
*Escherichia coli* O157:H7/*stx2*	1	0	0	0	0	0	0	1	1
*Escherichia coli* O157:H7/*eae*/*stx1*/*ehxA*	2	0	0	0	0	0	1	1	2
*Escherichia coli* O157	8	0	0	0	1	0	8	1	9
*Escherichia coli* O157/*eae*	1	0	0	0	0	0	0	1	1
*Escherichia coli* O157/*stx1*	1	0	0	0	0	0	0	1	1
*Escherichia coli* O157/*eae*/*ehxA*	1	1	0	0	0	1	0	1	2
*Escherichia coli* O157/*eae*/*stx1*/*stx2*/*ehxA*	1	0	0	0	0	0	0	1	1
*stx1*	1	0	0	0	0	0	1	0	1
stx2	1	0	0	0	0	0	0	1	1
*stx1*/*ehxA*	0	1	0	0	0	1	0	0	1
*eae*/*stx1*/*stx2/ehxA*	1	0	0	0	0	0	1	0	1
Total	22	4	0	1	1	3	12	13	28

The sum of RTE, processed, and raw meat samples is equal to the combined total positives for the different animal species.

*p*^a^ refers to processed meat (processed meat refers to nonintact meats such as ground/mince, patties, and sausage meats).

RTE, ready-to-eat.

Our findings revealed that the risk of contamination appeared to increase with processing, from raw meat to processed meat, which implies that there are other possible sources of contamination during processing of raw processed products. The raw meat could be exposed to contamination by contaminated workers’ hands and utensils or equipment such as meat mincing machines.

Although stringent regulations that are applied during further processing of RTE meats are expected to reduce the potential of contamination risk by STEC and non-STEC O157, vigilance is important during handling of RTE meats because these foods may be prone to recontamination and they are consumed without further processing.

In general, meat and meat products from cattle showed the highest number of positive samples (22/1758; 1.25%; CI 0.8–2), followed by poultry (4/1758; 0.23%; CI 0.1–1) and pork (1/1758; 0.06%); while no STEC strains were isolated from ovine meat (Table 2), which is not surprising as cattle are known to be natural reservoirs of STEC that remain asymptomatic (Padola and Etcheverria, 2014). Similar studies have been undertaken (Samadpour *et al.*, 2006; Ateba *et al.*, 2008; Rounds *et al.*, 2012; Magwedere *et al.*, 2013), but comparison with this study is challenging because of geographical differences, sample types or sample sizes, and the use of different diagnostic strategies (Dutta *et al.*, 2000; Ateba *et al.*, 2008; Magwedere *et al.*, 2013).

Table 2 shows a summary of the frequency of occurrence of different virulence factors (*eae*, *stx1*, *stx2*, and *ehxA*) that were tested in this study and their distribution among *Escherichia coli* O157:H7, *Escherichia coli* O157: non-H7, and non-O157 STEC isolates in meat from different animal species and meat types. Overall, 28/1758 (1.59%; CI 1.1–2) samples of meat and meat products from the South African domestic market tested positive for *Escherichia coli* O157:H7 and *Escherichia coli* O157: non-H7 (Table 3).

**Table 3.** Summary of the Virulence Gene Profiles of *Escherichia coli* O157:H7, *Escherichia coli* O157: Non-H7, and Non-O157 Shiga Toxin–Producing *Escherichia coli* Among Meat Samples from Different Animal Species and Meat Types for Positive Samples from South Africa

*E. coli* group	Virulence factor combinations				
	Eae	stx1	stx2	ehxA	Total^a^
*Escherichia coli* O157:H7 (*n* = 10)	+	−	−	+	1
	+	+	+	+	1
	+	−	−	−	3
	−	+	−	−	1
	+	−	+	−	1
	−	−	+	−	1
	+	+	−	+	2
*Escherichia coli* O157: non-H7 (*n* = 14)	−	−	−	−	9
	+	−	−	−	1
	−	+	−	−	1
	+	−	−	+	2
	+	+	+	+	1
Non-O157 STEC (*n* = 4)	−	+	−	−	1
	−	−	+	−	1
	−	+	−	+	1
	+	+	+	+	1
Total					28

*Escherichia coli* O157:H7 strains lacking all 4 virulence genes (*n* = 52) are not included in this table and fall outside the scope of this article.

^a^ Total refers to the total number of isolates with a specified combination of tested genes.

STEC, Shiga toxin–producing *Escherichia coli*.

*Escherichia coli* O157: non-H7 isolates were predominant (14/1758; 0.80%; CI 0.4–1), followed by *Escherichia coli* O157:H7 (10/1758; 0.57%; CI 0.3–1) and non-O157 STEC (4/1758; 0.23%; CI 0.1–1). The detection of *Escherichia coli* O157:H7 in different types of meats (Table 2) is an important finding because these pathogens have been linked to foodborne outbreaks (Browning *et al.*, 1990; Ito *et al.*, 1990; Bell *et al.*, 1994; Bosilevac and Koohmaraie, 2011). The significance of *Escherichia coli* O157:H7 is exacerbated by their low infective dose, severity of clinical manifestations, and case fatality rates (Lim *et al.*, 2010).

As contaminated cattle are the major sources of entry into the food value chain, it is important to implement an integrated risk-based approach from the farm level, during transportation and slaughter at abattoirs, and while handling in retail outlets up to the consumer level. Some of the *Escherichia coli* O157: non-H7 isolates did not possess *stx* genes. According to Ferdous *et al.* (2015), *stx*-encoding bacteriophage loss may happen during an infection or through culturing of strains, which may result in *Escherichia coli* O157 strains that lack *stx* virulence factors.

A total of 16 profiles were detected from *Escherichia coli* O157:H7; *Escherichia coli* O157: non-H7; and non-O157 STEC in this study (Table 2), and the highest variation of strains was observed from beef. Two of the isolates had the profile *Escherichia coli* O157:H7/*eae*/*stx_1_*/*stx_2_*/*ehxA* (1 poultry) and *Escherichia coli* O157/*eae*/*stx_1_*/*stx_2_*/*ehxA* (1 beef). The profiles of 3 non-O157 STEC isolates that were detected in beef samples were *eae*/*stx_1_/stx_2_*/*ehxA*, *stx_1_*, and *stx_2_* and the pattern from poultry was *stx_1_*/*ehxA* (Table 2).

The diverse genetic profiles of *Escherichia coli* O157:H7; *Escherichia coli* O157: non-H7; and non-O157 STEC from this study demonstrate wide variation in population structure and characteristics of these potential pathogens, which improves the knowledge about possible pathogenicity and risks to human health. As more variants are possible, it is important to continue undertaking surveillance studies to improve understanding of strains that are circulating in South Africa as part of emergency preparedness and to avoid potential foodborne outbreaks.

In this study, four virulence factors (*stx1*, *stx2*, *eae*, and *hlyA*) were analyzed and observed 37 times (*n* = 9 *stx_1_*; *n* = 6 *stx_2_*; *n* = 13 *eae*; and *n* = 9 *ehxA*; Table 3). Shiga toxins are known to be important for STEC virulence in human beings and may cause severe gastroenteritis (Gonzalez-Escalona and Kase, 2019). The *eae* gene is an important virulence factor because it produces intimin, which has a role in the formation of attaching and effacing lesions (Yang *et al.*, 2020), hence some strains from this study have the possibility of being pathogenic to humans.

Infection with strains that lack *eae* genes poses relatively less risk of developing serious complications such as HUS (Yang *et al.*, 2020). Tables 2 and 3 show some *Escherichia coli* O157:H7 strains from this study with both *stx* and *eae*, which make them potential enterohemorrhagic *E. coli* (EHEC) (Chahed *et al.*, 2006). The EHEC are known to cause bloody diarrhea and it is important to undertake risk-based studies of these pathogens along the meat value chain in South Africa. The detection of non-O157 STEC was much lower (*n* = 4) compared with *Escherichia coli* O157:H7 (Table 3), but the presence of these bacteria in meat and meat products should not be ignored because they are considered as emerging pathogens (Dutta *et al.*, 2000).

Four of the 259 (2.0%) imported meat and meat product samples from ports of entry tested positive for *Escherichia coli* O157:H7 based on the MicroSEQ *Escherichia coli* O157:H7 Detection Kit (Applied Biosystems; Thermo Fisher Scientific), but these bacteria could be isolated from only 2 (1.0%) of the samples. All of the positive samples were from raw poultry. Three of the four *Escherichia coli* O157:H7 isolates were devoid of the four tested virulence genes, but one isolate carried all four tested virulence factors (*stx_1_*, *stx_2_*, *eae*, and *ehxA*).

In this study, there was a bias toward collection of poultry samples at the ports of entry compared with ruminant meat samples. The fewer ruminant samples collected at the ports of entry compared with the samples collected on the domestic market could have led to the observed variations in incidence between imported and domestic samples. The number of imported samples was dependent on availability.

Related studies where abattoir beef trims were screened for the presence of Shiga toxin (*stx*_1_ and *stx*_2_) and intimin (*eae*) virulence genes in Namibia showed that 136/771 (17.64%) samples were positive for both *stx* and *eae* virulence genes, with nine positive for O26, O45, O103, O111, and O121 and three for O145 (Molini *et al.*, 2016).

The number of isolates that were recovered from the domestic South African market by culture were fewer (*n* = 17/1758; 0.97%) compared with 28/1758 positive samples that were detected by RT-PCR (1.59%). This implies that the RT-PCR detection rate was ∼1.64 times compared with culture-based methods. It may be plausible to assume that the low storage temperature of samples could have contributed to the low isolation rate, but even so, it is likely that the differences in detection rates could possibly be explained by different sensitivities of the methods.

All culture-positive isolates were RT-PCR positive. It becomes important to use both RT-PCR and culture-based techniques to avoid false-negative results that could result in contaminated meat entering the meat value chain and/or false-positive results that could result in unnecessary condemnation of food (Arthur *et al.*, 2004). Singh and Mustapha, 2015; Kanankege *et al.*, 2017. The relatively lower isolation rate has been attributed to the influence of background flora compared with small numbers of STEC strains in foods and phages in meat that carry *stx* among other reasons (Ju *et al.*, 2012).

Continuous improvement and optimization of culture-based methods for isolation of STEC are recommended as they may be important for risk-based studies and understanding population structure, characteristics, pathogenicity, and the extent of potential disease severity of these pathogens.

### Limitations of the study

Phenotypic serotyping of four non-O157 STEC isolates was outside the scope of this study. The sample size for imported meat was not representative of the volumes imported into the country for each species category. The range of sample sizes for the different animal species was large (from 33 for game meat to 1077 for beef), hence interpretation and discussion of the findings are done based on the proportion of positive samples.

A relatively large number (*n* = 52) of incidental *Escherichia coli* O157:H7 strains lacking all virulence factors (*eae*, *stx1*, *stx2*, and *ehxA*) were observed, but these were not characterized in detail to warrant further discussion in this article. However, such isolates may be important in future.

## Conclusions

This study highlighted the presence and virulence factors of *Escherichia coli* O157, *Escherichia coli* O157:H7, and non-STEC O157 in raw, processed (nonintact), and RTE meat products in South Africa, with processed (nonintact) meat and RTE showing the highest number of positive samples. This is important from a “One Health” standpoint due to the complex nature of meat contamination along the entire value chain.

Contamination of RTE meat is noteworthy because of the potentially high risk of infections as no further processing is required before consumption and the possible risk of recontamination. Despite the use of two different types of culture media, RT-PCR was more sensitive compared with culture, hence development of an efficient model that can be used for routine diagnosis of STEC and non-STEC O157 from meat is important for accurate diagnosis of STEC contamination in food.

Future research should focus on next-generation sequencing of STEC and non-STEC O157 isolates to better understand the epidemiology and population structure of these pathogens.
